# Simvastatin Resistance of *Leishmania amazonensis* Induces Sterol Remodeling and Cross-Resistance to Sterol Pathway and Serine Protease Inhibitors

**DOI:** 10.3390/microorganisms10020398

**Published:** 2022-02-09

**Authors:** Thais Tenorio Soares Fujii, Pollyanna Stephanie Gomes, Rubens Lima do Monte-Neto, Daniel Claudio de Oliveira Gomes, Marc Ouellette, Eduardo Caio Torres-Santos, Valter Viana Andrade-Neto, Herbert Leonel de Matos Guedes

**Affiliations:** 1 Laboratório de Imunofarmacologia, Instituto de Biofísica Carlos Chagas Filho (IBCCF), Universidade Federal do Rio de Janeiro, Rio de Janeiro 21941-901, Brazil; thaistenoriosoares@gmail.com; 2 Laboratório de Imunobiotecnologia, Instituto de Microbiologia Paulo de Góes, Universidade Federal do Rio de Janeiro, Rio de Janeiro 21941-901, Brazil; pollyannaufrj@gmail.com; 3 Laboratório Interdisciplinar de Pesquisas Médicas, Instituto Oswaldo Cruz, Fundação Oswaldo Cruz-Fiocruz, Rio de Janeiro 21040-900, Brazil; 4 Grupo de Pesquisas em Biotecnologia Aplicada a Patógenos—Instituto René Rachou, Fundação Oswaldo Cruz-Fiocruz Minas, Belo Horizonte 30190-002, Brazil; rubens.monte@fiocruz.br; 5 Núcleo de Doenças Infecciosas, Universidade Federal do Espírito Santo, Vitória 29075-910, Brazil; dgomes@ndi.ufes.br; 6 CHU de Quebec Research Center, Division of Infectious Disease and Immunity, Department of Microbiology Infectious Disease and Immunology, Laval University, Quebec, QC G1V 4G2, Canada; marc.ouellette@crchudequebec.ulaval.ca; 7 Laboratório de Bioquímica de Tripanosomatídeos, Instituto Oswaldo Cruz, Fundação Oswaldo Cruz-Fiocruz, Rio de Janeiro 21040-900, Brazil; ects@ioc.fiocruz.br

**Keywords:** *Leishmania amazonensis*, sterol pathway, HMG-CoA reductase, simvastatin resistance, pharmacological target, serine proteases

## Abstract

The sterol biosynthesis pathway of *Leishmania* spp. is used as a pharmacological target; however, available information about the mechanisms of the regulation and remodeling of sterol-related genes is scarce. The present study investigated compensatory mechanisms of the sterol biosynthesis pathway using an inhibitor of HMG-CoA reductase (simvastatin) and by developing drug-resistant parasites to evaluate the impact on sterol remodeling, cross-resistance, and gene expression. Simvastatin-resistant *L. amazonensis* parasites (*La*SimR) underwent reprogramming of sterol metabolism manifested as an increase in cholestane- and stigmastane-based sterols and a decrease in ergostane-based sterols. The levels of the transcripts of sterol 24-C-methyltransferase (SMT), sterol C14-α-demethylase (C14DM), and protease subtilisin (SUB) were increased in *La*SimR. *La*SimR was cross-resistance to ketoconazole (a C14DM inhibitor) and remained sensitive to terbinafine (an inhibitor of squalene monooxygenase). Sensitivity of the *La*SimR mutant to other antileishmanial drugs unrelated to the sterol biosynthesis pathway, such as trivalent antimony and pentamidine, was similar to that of the wild-type strain; however, *La*SimR was cross-resistant to miltefosine, general serine protease inhibitor *N*-*p*-tosyl-l-phenylalanine chloromethyl ketone (TPCK), subtilisin-specific inhibitor 4-[(diethylamino)methyl]-*N*-[2-(2-methoxyphenyl)ethyl]-*N*-(3R)-3-pyrrolidinyl-benzamide dihydrochloride (PF-429242), and tunicamycin. The findings on the regulation of the sterol pathway can support the development of drugs and protease inhibitors targeting this route in parasites.

## 1. Introduction

Parasites of the Trypanosomatidae family, such are *Trypanosoma cruzi* and *Leishmania* spp., which are the causative agents of Chagas disease and leishmaniasis, respectively, produce ergosterol and other sterols with methylation at carbon-24 [1]. Sterols with the cholestane skeleton are methylated by the enzyme sterol 24-C-methyltransferase (SMT) in one of the final steps of the sterol biosynthesis pathway (SBP); SMT catalyzes the transfer of the methyl group from S-adenosyl methionine (SAM) to the carbon-24 position of the sterol side chain. This reaction does not occur in mammalian cells due to the absence of SMT [2]. Since the SMT reaction is parasite-specific, this part of the SBP is a potential target for chemotherapy of leishmaniasis. This application has been assessed in extensive studies of several pathway inhibitors, such as statins (simvastatin), allylamines (terbinafine), azoles (ketoconazole), and azasterols. Pharmacological inhibition at various stages of the SBP results in parasite death, confirming that the SBP is an essential biochemical pathway [3,4,5,6,7,8,9,10,11].

The enzyme 3-hydroxy-3-methylglutaryl-coenzyme A (HMG-CoA) reductase (HMGR) catalyzes the conversion of HMG-CoA to mevalonate, a precursor of cholesterol and other isoprenoids in humans, and to ergosterol in fungi and *Leishmania* [12,13]. Statins are important inhibitors of HMGR, which is an important enzyme catalyzing the rate-limiting step of the cholesterol biosynthesis pathway; statins are lipid-lowering drugs used to treat hypercholesterolemia and dyslipidemia and are considered beneficial for the prevention of cardiovascular events [14].

Statins (simvastatin, atorvastatin, and mevastatin) potently interfere with the growth of protozoan parasites of the Trypanosomatidae family by inhibiting HMGR and decreasing the ergosterol level [15,16,17]. HMGR was proposed as a potential drug target in *L. donovani* [18]. However, the impact of statins on sterol metabolism and gene expression in *Leishmania* is unknown.

Cholesterol is a crucial component of the cell membrane of mammalian cells, and cholesterol biosynthesis is highly regulated by a feedback mechanism to maintain the appropriate levels of this molecule [19]. In the absence of cholesterol, site-1 protease (S1P) (also known as MBTP1), which is a member of the subtilisin (SUB) serine protease family S8A subfamily, acts in combination with other proteins to increase gene transcription and sterol biosynthesis [20]. The expression of S1P (subtilisin-related protease) is increased in mammalian cells at low levels of sterols [21].

The sequence of the *Leishmania* SUB gene has been determined; the catalytic region of SUB is relatively conserved among *Leishmania* species, and their genome contains only a single copy of *SUB* [22]. Similar to mammalian S1P, *Leishmania* SUB is a serine protease of the S8A subfamily [23]. Subtilisin promotes the survival of *Leishmania* amastigotes, serving as the terminal processor of peroxidase of the trypanothione reductase system, assisting in redox homeostasis and protecting the parasite from oxidative stress within host macrophages [22].

The ergosterol biosynthesis pathway has been exploited as a pharmacological target, and understanding the modulation and regulation of this pathway is important for the development of new drugs for the treatment of leishmaniasis. The modulation of metabolic pathways can be studied by induction of resistance using drugs that inhibit specific enzymes of the pathway [24]. Information about the mechanisms of resistance against anti-*Leishmania* drugs can also be derived from the parasites with resistance induced in vitro, mainly in their promastigote forms [25]. The induction of resistance in vitro in *Leishmania* spp. and *T. cruzi* has been extensively studied, including resistance to sterol biosynthesis inhibitors, such as azoles [26,27,28].

We selected *Leishmania* parasites resistant to HMGR inhibitor simvastatin to investigate the mechanism and effect of the resistance on the parasite. HMGR is an enzyme that functions at the beginning of the sterol pathway. The results of the present study indicated that simvastatin-resistant *L. amazonensis* (*La*SimR) had an altered sterol profile. Specifically, *La*SimR parasites accumulated cholestane- and stigmastane-based sterols, manifested an increase in the levels of SUB, C14DM, and SMT mRNAs, and were cross-resistant to ketoconazole, a C14-α-demethylase inhibitor, and to two serine protease inhibitors. These results suggest that serine proteases may be involved in sterol regulation in the parasite.

## 2. Materials and Methods

### 2.1. Parasites

Promastigotes of *Leishmania amazonensis* (MHOM/BR/77/LTB 0016) were maintained at 26 °C in RPMI medium (Sigma-Aldrich Inc., St. Louis, MO, USA) supplemented with 10% fetal bovine serum (FBS), 100 μg/mL streptomycin, 100 U/mL penicillin, 5 mg/mL hemin, 0.5 mg/mL folic acid, 0.2 mg/mL D-biotin, and 4 mg/mL adenine. Parasites were obtained from the lesions of BALB/c mice, were maintained in culture, and passaged twice a week.

### 2.2. Animals

Male BALB/c mice 10–12 weeks of age (weighing approximately 20 g) were obtained from the Institute of Science and Technology in Biomodels (ICTB/FIOCRUZ). All experiments were performed following the guidelines and regulation of the Brazilian National Council for the Control of Animal Experimentation and were approved by the Ethics Committee on Animal Use of Instituto Oswaldo Cruz (CEUA/IOC, license L-026/2015-A5). This study was compliant with the ARRIVE requirements.

### 2.3. Selection of LaSimR

*L. amazonensis* promastigotes were cultured in RPMI medium, and the *La*SimR parasites were selected in a stepwise selection process using simvastatin (Sim) concentrations increasing from 20 to 75 μM. The process took approximately 4 months of the passage of both Sim-treated and wild-type non-treated cultures. Selected resistant parasites were maintained under drug pressure (75 μM Sim). We induced resistance in three independent cultures. After resistance induction, we derived three clones from each flask by plating the population of resistant strain on SDM-agar (1% Noble Agar, Nunc) containing 75 μM of simvastatin [29,30]. The experiments were performed with the three clones derived from independent cultures (each culture was tested in triplicate), and the data were plotted as their mean and standard error of the mean (SEM). Parasite growth was monitored by light microscopy, and the parasites were passaged at an initial inoculum of 1 × 10^6^ promastigotes/mL. The growth curve was assessed at an initial inoculum of 5 × 10^5^ promastigotes/mL by counting in a Neubauer chamber every 24 h for 12 days.

### 2.4. Antileishmanial Activity

*L. amazonensis* promastigotes (1 × 10^6^) were incubated at 26 °C for 72 h in RPMI medium without phenol red (Sigma-Aldrich Inc., St. Louis, MO, USA), pH 6.5, and treated as described below to evaluate the leishmanicidal activity of various drugs. Wild-type (WT; *La*WT) and *La*SimR parasites were washed and cultured (I) without Sim, (II) with inhibitors of ergosterol biosynthesis (0–256 μM in the case of Sim), and (III) with 0–128 μM terbinafine and ketoconazole (Sigma-Aldrich). Concentrations of the reference drugs varied from 0 to 48 μM for miltefosine, from 0 to 100 μM for trivalent antimony (Sb^III^), and from 0 to 3 μM for pentamidine (Sigma-Aldrich). Serine protease inhibitors were used at the following concentrations: from 0 to 256 μM for N-p-tosyl-L-phenylalanine chloromethyl ketone (TPCK) and from 0 to 128 μM for PF-429242 (PF; 4-[(diethylamino)methyl]-*N*-[2-(2-methoxyphenyl)ethyl]-*N*-(3R)-3-pyrrolidinyl-benzamide dihydrochloride) (Sigma-Aldrich). The concentrations of tunicamycin, an inhibitor of bacterial and eukaryotic *N*-acetylglucosamine transferases, were from 0 to 16 μM. Parasite viability was assessed using 50 μM resazurin (alamarBlue^®^) for 4 h at 26 °C. The reaction product was measured by fluorimetry (SpectraMax Gemini XPS, Molecular Devices, San Jose, CA, USA) (excitation at 560 nm and emission at 590 nm). The assays were performed in triplicate, and the experiments were repeated three times. The values of the parasite growth were calculated as percentages relative to the untreated control samples.

### 2.5. Lipid Extraction

The lipids were extracted from *La*WT and *La*SimR promastigotes using the method of Bligh and Dryer (1959) [31]. The initial concentration of the parasites was 1 × 10^6^ promastigotes/mL, and the samples were incubated for 48 h (*La*WT and *La*SimR), 72 h (*La*WT, *La*SimR, and *La*SimR + 75 μM Sim), and 288 h (or 12 days) (*La*SimR + 75 μM Sim). Then, a mixture of methanol, chloroform, and water (1:0.5:0.4 *v*/*v*) was added to the samples. The mixture was agitated every 5 min for 1 h and centrifuged at 1000× *g* for 20 min; the resulting supernatant containing lipids was separated from the precipitate. The precipitate was subjected to second extraction using the same procedure. The supernatants from both extractions were pooled, and a mixture of distilled water and chloroform (1:1 *v*/*v*) was added. The samples were vortexed for 20 s, and the mixture was centrifuged at 1000× g for 30 min. The lower (organic) phase containing lipids was separated using a glass syringe and transferred to 1.5 mL glass tubes resistant to organic solvents (Axygen Scientific, Inc., Union City, CA, USA). The solvent was evaporated under the flow of nitrogen gas (N_2_), and the lipids were analyzed by gas chromatography coupled to mass spectrometry (GC-MS).

### 2.6. Sterol Profile Analysis by GC-MS

GC-MS was used to separate and identify sterols. The samples were injected into a GC-MS-QP2010 Ultra system (Shimadzu Scientific Instruments, Tokyo, Japan). After the injection, column temperature (RTX-SMS column type) was maintained at 50 °C for 1 min, increased to 270 °C at 10 °C/min, and increased to 300 °C at 1 °C/min. The gas flow (He) was maintained constant at 1.1 mL/min. Injector and detector temperatures were 250 and 280 °C, respectively [32].

### 2.7. Serum Delipidation and Analysis of Neutral Lipids by Thin Layer Chromatography (TLC)

Serum delipidation was performed as described previously [33]. FBS aliquots containing 0.1 mg/mL EDTA (to prevent interaction with traces of peroxide from the solvent) were added to 10 mL of butanol and DIPE (diisopropyl ether) mixture (40:60, *v*/*v*) (Tedia, Brazil). The tubes were then end-over-end rotated at 28–30 rpm for 30 min. The mixture was centrifuged at 500× *g* for 2 min to separate the aqueous and organic phases. The aqueous phase was carefully removed by suction with a needle and syringe, and the traces of the solvent were eliminated under a stream of N_2_. Delipidation was confirmed by TLC (Supplementary Figure S1). Delipidated FBS (FBSd) was then sterilized by membrane filtration through a 0.22 µm Millex-GV filter (Millipore SA, Molsheim, France).

Sterols obtained from FBSd were dissolved in chloroform and analyzed by TLC on a silica plate (Silica Gel 60 F254, Merck, Frankfurt, Germany). The plate was previously impregnated with silver nitrate (1%) in methanol to enhance the separation of the lipids with double bonds, particularly the separation of ergostane-related sterols from cholesterol. The plate was developed in two steps. The first step was developed in hexane:ethyl ether:acetic acid (60:40:1, *v*/*v*), and the second step was developed in hexane:chloroform:acetic acid (80:20:1, *v*/*v*). The spots were detected using charring reagent (CuSO_4_) and heating at 200 °C for 20 min [34].

### 2.8. Extraction of mRNA

*La*WT and *La*SimR parasites were cultivated for 48 and 72 h, and *La*SimR + 75 μM Sim was cultivated for 72 and 288 h (12 days). Parasites were homogenized in 500 μL of TRIzol™ reagent (Sigma-Aldrich Inc., St. Louis, MO, USA) to stabilize the RNA. RNA was extracted by adding 100 μL chloroform to the tubes, which were vigorously shaken for 20 s. Then, the samples were incubated for 3 min at room temperature and centrifuged for 10 min at 4 °C at maximum speed. The aqueous phase was collected, and absolute ethanol was added at a 1:1 ratio. Subsequent steps were performed using a RNeasy Mini kit (Qiagen, Hilden, Germany) according to the manufacturer’s instructions. RNA concentration was determined using a NanoDrop Lite spectrophotometer (Thermo Fischer Scientific, Waltham, MA, USA). Complementary DNA synthesis was performed using a SuperScript™ III first-strand synthesis SuperMix kit (Thermo Fischer Scientific, Waltham, MA, USA) according to the manufacturer’s instruction. Each synthesis sample used 3 μg of RNA. Promastigotes of *L. amazonensis* were cultured in the presence of FBSd or FBS for 12 h to assess gene expression in the absence of exogenous cholesterol. After incubation, RNA was extracted according to the same protocol.

### 2.9. Transcript Levels in L. amazonensis Promastigotes

Power SYBR^®^ Green PCR Master Mix (Thermo Fischer Scientific, Waltham, MA, USA) was used in a StepOne™ real-time PCR thermal cycler system (Thermo Fischer Scientific, Waltham, MA, USA) for quantitative real-time PCR (RT-qPCR). cDNA concentration was quantified using a Qubit^®^ ssDNA assay kit (Life Technologies, Eugene, Oregon, USA) and adjusted to a final concentration of 10 ng/μL. Equivalent amounts of cDNA in triplicate were amplified in a total volume of 12.5 μL containing 6.25 μL of Power SYBR^®^ Green PCR Master Mix, 0.15 μL of the forward primer (0.3 μM), 0.15 μL of the reverse primer (0.3 μM), 1 μL of cDNA, and 4.95 μL of nuclease-free water. The following thermal cycler program was used: initial incubation at 95 °C for 10 min and 40 cycles at 95 °C for 15 s and at 60 °C for 1 min. The relative amount of the generated PCR products for each primer set was measured as the threshold (C_t_) value, and the results were calculated using the 2^−ΔΔCt^ method; glyceraldehyde 3-phosphate dehydrogenase (GAPDH) gene was used as an endogenous control. The gene expression levels were normalized by analyzing constitutive mRNA expression. The primer sequences were as follows: the HMGR gene: forward, 5′-GCGGAGGCCGTCATCA-3′ and reverse, 5′-TCCACCGTGCACTTGAGAAC-3′; the C14DM gene: forward, 5′-CCCGCGTAACGAGATTCTCT-3′ and reverse, 5′-CAAAGACGGGCACCATGAAC-3′; the SUB gene: forward, 5′-TGGATGTGATTAGCCTGTCCTATG-3′ and reverse, 5′-CTC ATGCATCAGCCGGTACA-3′; the SMT gene: forward, 5′-ATGAGCTTAGCCGACAAC AC-3′ and reverse, 5′-GGGCTTGATGACACGAAAGA-3′; the actin gene: forward, 5′-GCTGTGTTGTCCCTGTACTC-3′ and reverse, 5′-AGCGAGTAACCCTCGTAGAT-3′; the GAPDH gene (endogenous control); forward, 5′-GGTAAGCTCGGTGTGGATTAC-3′ and reverse, 5′-CGCTGATCACGACCTTCTTC-3′; and the squalene monooxygenase gene: forward, 5′-GCTGAAAGAGGTAGGCATGAA-3′ and reverse, 5′-CTTATCGTCCACCACCACATAG-3′.

### 2.10. Intracellular Accumulation of Rhod-123 in LaWT and LaSimR

The efflux of the fluorescent probe Rhod-123 (Sigma-Aldrich Inc., St. Louis, MO, USA) was tested in the *La*WT and *La*SimR strains using a cytometer (CytoFLEX S Beckman Coulter). First, promastigotes of *L. amazonensis* (5 × 10^6^ parasites/mL) in the log-phase of growth were incubated in RPMI medium in the presence and the absence of 100 μM of verapamil hydrochloride (Vp) (Sigma-Aldrich Inc., St. Louis, MO, USA), an inhibitor of the drug efflux pump P-glycoprotein, for 1 h at 26 °C. Next, parasites were incubated in the presence or absence of Rhodamine-123 (Rhod-123) (5 μg/mL) for 30 min [35,36]. The parasites were then washed three times, resuspended in 1 mL PBS buffer, and incubated in the presence or absence of Vp (100 μM) for 30 min and 90 min, in triplicates, to measure Rhod-123 efflux. Data analysis was performed using the CytExpert software.

### 2.11. Determination of the Parasite Resistance after Exposure to H_2_O_2_

To determine the EC_50_ for H_2_O_2_, *L. amazonensis* promastigotes *La*WT and *La*SimR (1 × 10^6^ parasites/mL) were incubated at 26 °C for 72 h in RPMI medium without phenol red (Sigma-Aldrich), pH 6.5, and exposed to H_2_O_2_ (0–6400 μM) [37]. Parasite viability was assessed using 50 μM resazurin (alamarBlue^®^) for 4 h at 26 °C. The reaction product was measured by fluorimetry (SpectraMax Gemini XPS, Molecular Devices, San Jose, CA, USA) (excitation at 560 nm and emission at 590 nm). The assays were performed in triplicate, and the experiments were repeated three times. The values of the parasite growth were calculated as percentages relative to the untreated control samples.

### 2.12. Statistical Analysis

Statistical analyses were performed using GraphPad Prism 6 software (La Jolla, San Diego, CA, USA). Statistical differences between the mean values were evaluated by two-way ANOVA with Sidak multiple comparisons test to determine the significance of the differences. The results are expressed as the mean ± standard error of the mean (SEMs), and differences between the control and treated groups were considered statistically significant when *p* ≤ 0.05. The EC_50_ values were obtained by nonlinear regression using GraphPad Prism 6 software.

## 3. Results

### 3.1. Simvastatin-Induced Resistance of L. amazonensis

**Figure 1:**
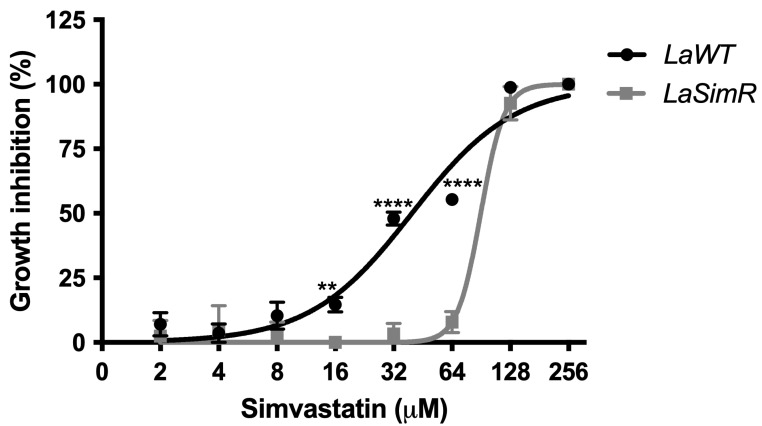
Leishmanicidal activity of simvastatin in the wild-type and simvastatin-resistant strains of *L. amazonensis*. Wild-type (*La*WT) and simvastatin-resistant (*La*SimR) promastigotes were incubated at an initial concentration of 1 × 10^6^ promastigotes/mL in the presence of various concentrations of simvastatin (0–256 μM) in 96-well plates for 72 h at 26 °C. After incubation, the growth was evaluated using resazurin (alamarBlue^®^); after 4 h, the reaction was evaluated by fluorimetry (excitation at 560 nm and emission at 590 nm). The experiments were performed in triplicate and repeated three times. ** *p* < 0.01; **** *p* < 0.0001.

Resistance was induced stepwise in *L. amazonensis* promastigotes until obtaining three independent cultures resistant to 75 μM simvastatin (*La*SimR). All experiments were conducted with the clones derived from each resistant culture (Supplementary Figure S2). Growth curves demonstrated similar profiles for *La*WT, *La*SimR, although *La*SimR in the presence of 75 μM simvastatin exhibited a growth defect (Supplementary Figure S3). *La*WT parasites were sensitive to simvastatin, with a mean EC_50_ of 39.72 μM (95% CI: 33.47–47.15 μM) (Figure 1) whereas the *La*SimR parasites showed a 2.3-fold increase in resistance with a mean EC_50_ of 90.04 μM (95% CI: 85.46–94.87 μM).

### 3.2. Different Sterol Profiles of Simvastatin-Resistant Parasites

The content of sterols in *La*WT and *La*SimR parasites was analyzed by GC-MS at different times. The data of Table 1 indicate that the relative amount of cholesterol (1) was similar in these two strains, independent of the culture time. The ergostane-derived sterols were decreased in the *La*SimR strain compared to the *La*WT strain (6 and 7; Table 1), which was accompanied by an increase in cholesta-5,7,24-trien-3β-ol (4) and stigmasta-5,7,22-trien-3β-ol (5), a minor constituent (48 h of incubation). Interestingly, with the culture progression (72 h of incubation), the *La*WT continues synthesizing dehydroepisterol (6), the major sterol of *L. amazonensis*, and the *La*SimR strain decreases its content and accumulates cholesta-5,7,24-trien-3β-ol (4), stigmasta-5,22-dien-3β-ol (3), and stigmasta-5,7,22-trien-3β-ol (5). Following, we analyzed how the simvastatin treatment impacts the sterol content in the resistant strain. *La*SimR has grown up with simvastatin 75 µM for 72 h reduced to nearly undetectable levels its content in dehydroepisterol (6), which was maintained after 288 h of drug pressure. Unexpectedly, the content of the stigmastane-based sterol (5) increased under the simvastatin treatment, while the level of cholesta-5,7,24-trien-3β-ol (4) decreased from 72 to 288 h of incubation.

### 3.3. Transcript Levels in LaSimR

**Figure 2:**
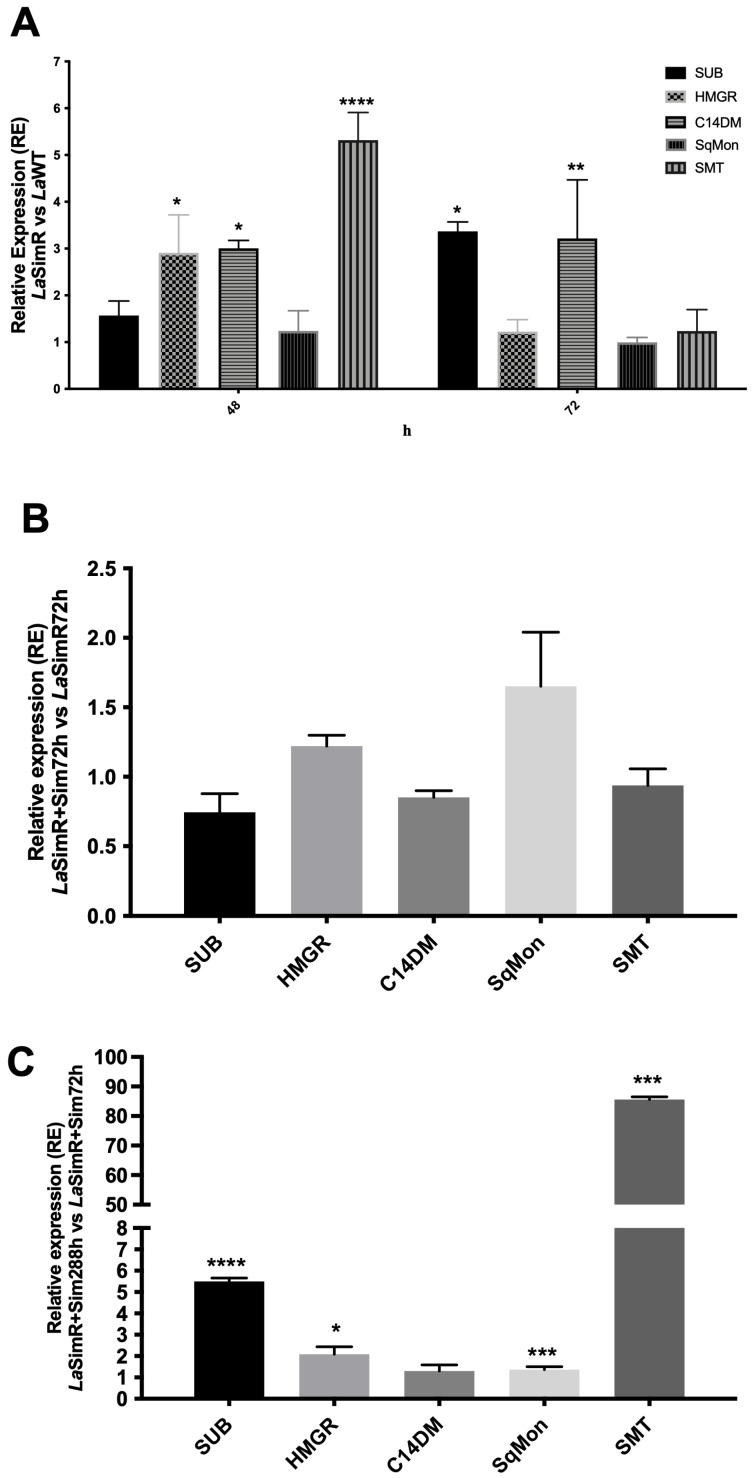
Gene regulation in the simvastatin-resistant strain of *L. amazonensis*. The levels of subtilisin (SUB), HMG-CoA reductase (HMGR), C14-demethylase (C14DM), squalene monooxygenase (SqMon), and sterol 24-C-methyltransferase (SMT) transcripts were evaluated by real-time (quantitative) PCR in wild-type (*La*WT) and simvastatin-resistant (*La*SimR) promastigotes. The expression of glyceraldehyde 3-phosphate dehydrogenase (GAPDH) gene was used to normalize the data. (**A**) Relative expression in *La*SimR vs. *La*WT cultured for 48 and 72 h; (**B**) relative expression in *La*SimR + Sim 72 h vs. *La*SimR 72 h; and (**C**) relative expression in *La*SimR + Sim 288 h vs. *La*SimR + Sim 72 h. The experiments were performed in triplicate and repeated three times. * *p* < 0.05; ** *p* < 0.01; *** *p*< 0.001; **** *p* < 0.0001.

The sterol profile of *La*SimR parasites cultured in the presence or in the absence of simvastatin was characterized by an increase in cholestane and stigmata-related sterols. SMT enables the biosynthesis of stigmata-related sterols; hence, we evaluated the levels of the transcripts of the SMT gene. Changes in mRNA levels encoding proteins involved in sterol biosynthesis, including HMGR, C14DM, squalene monooxygenase (SqMon), and SMT, in *La*SimR parasites were evaluated and compared to those in the *La*WT strain (Figure 2). The levels of the transcript of the *Leishmania* SUB-encoding gene were also analyzed.

The ΔΔC_t_ values in the *La*SimR strain cultivated for 48 h indicated a significant accumulation of mRNA of HMGR, three-fold greater expression of C14DM, and five-fold greater expression of SMT compared with those in the *La*WT strain (Figure 2A). After 72 h of growth, the levels of subtilisin transcripts in the *La*SimR strain were 3.5-fold higher than that in the *La*WT strain. The accumulation of C14DM transcripts was maintained in the resistant strain; however, the levels of the HMGR transcript were similar (Figure 2A). Additionally, the data of Figure 2B indicated that the levels of SMT mRNA were similar between *La*SimR + Sim (at 288 h) compared with that in the *La*SimR strain (at 72 h). However, a comparison of *La*SimR + Sim cultivated for either 12 days or 72 h indicated an 80-fold increase in SMT, a 6-fold increase in the accumulation of SUB, and an increase in HMGR (Figure 2C).

**Figure 3:**
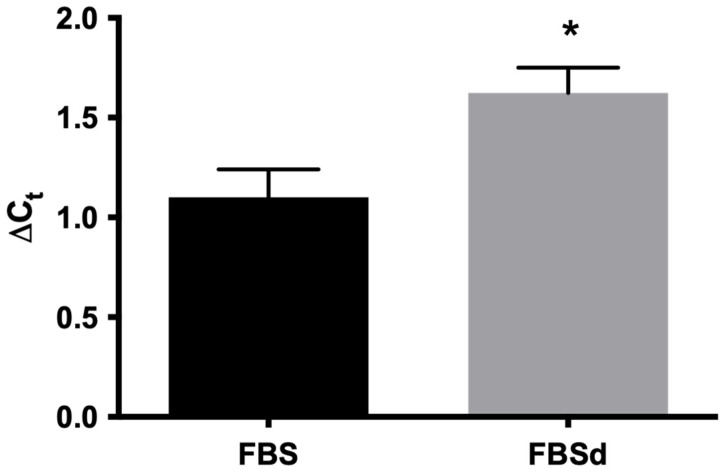
Expression of subtilisin mRNA in *L. amazonensis* in the presence of FBS and FBSd. *L. amazonensis* promastigotes were cultivated in RPMI medium containing 10% FSB or FSBd for 72 h. The expression of subtilisin mRNA was evaluated by real-time PCR. The expression of the glyceraldehyde 3-phosphate dehydrogenase (GAPDH) gene was used to normalize the data. The experiments were performed in triplicate and repeated three times. * *p* < 0.05.

Intrigued by our observation in the alteration of subtilisin gene expression in simvastatin-resistant parasites (Figure 2) with altered levels of sterols (Table 1), we also evaluated the effect of cholesterol deprivation on subtilisin expression in *La*WT parasites. Growth of promastigotes of *L. amazonensis* in the presence of delipidated serum for 72 h resulted in an increase in the expression of the subtilisin gene (Figure 3). In mammalian cells, subtilisin is regulated by sterols [20,21], so these results reinforce the possible relationship between SUB and sterol biosynthesis.

### 3.4. Cross-Resistance to Ergosterol Biosynthesis Inhibitors

**Figure 4:**
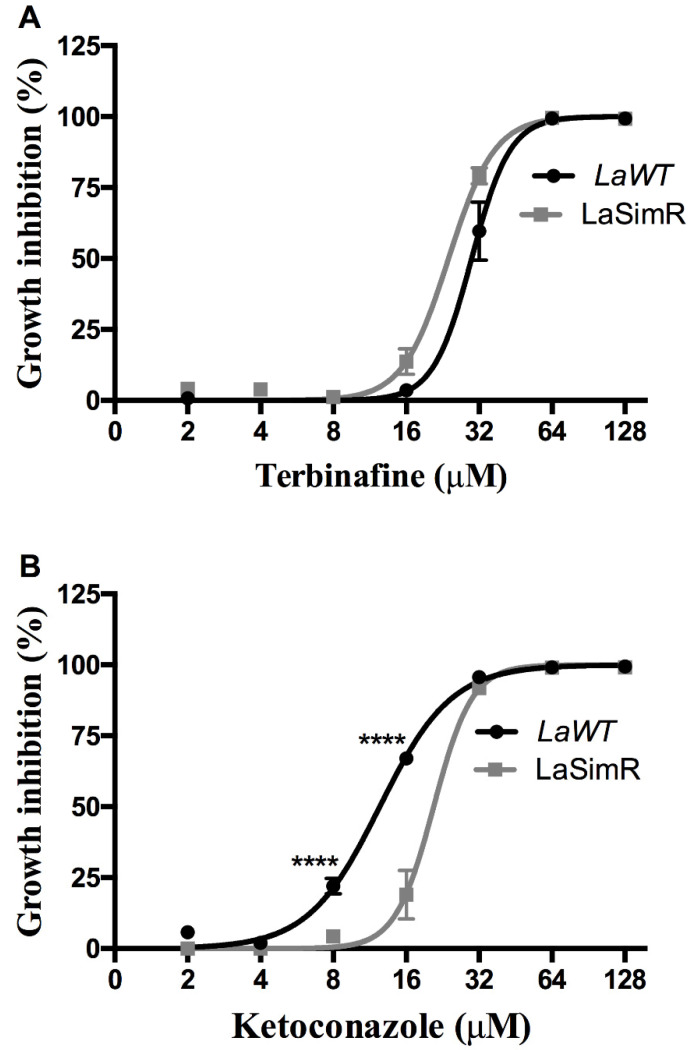
Leishmanicidal activity of terbinafine and ketoconazole in the wild-type and simvastatin-resistant strains of *L. amazonensis*. Wild-type (*La*WT) and simvastatin-resistant (*La*SimR) promastigotes were incubated at an initial concentration of 1 × 10^6^ promastigotes/mL in the presence of indicated concentrations of terbinafine (0–128 μM) or ketoconazole (0–128 μM) for 72 h at 26 °C. After incubation, the growth was evaluated using resazurin (alamarBlue^®^); after 4 h, the reaction was evaluated by fluorimetry (excitation at 560 nm and emission at 590 nm). (**A**) Terbinafine and (**B**) ketoconazole. The experiments were performed in triplicate and repeated three times. **** *p* < 0.0001.

To determine whether resistance to simvastatin influences various steps of ergosterol biosynthesis, we evaluated cross-resistance to terbinafine, a squalene epoxidase inhibitor, and ketoconazole, a C14DM inhibitor (Table 2). Both *La*WT and *La*SimR strains were sensitive to terbinafine, with the EC_50_ values of 29.77 μM (95% CI: 27.79–31.89 μM) and 23.90 μM (95% CI: 22.78–25.08 μM), respectively (Figure 4A). However, treatment with various concentrations of ketoconazole revealed cross-resistance of the *La*SimR strain, with the mean EC_50_ values of 12.43 μM (95% CI: 11.84–13.05 μM) in *La*WT and 20.76 μM (95% CI: 19.29–22.34 μM) in *La*SimR (Figure 4B).

### 3.5. Evaluation of Cross-Resistance to Current Drugs against Leishmaniasis

**Figure 5:**
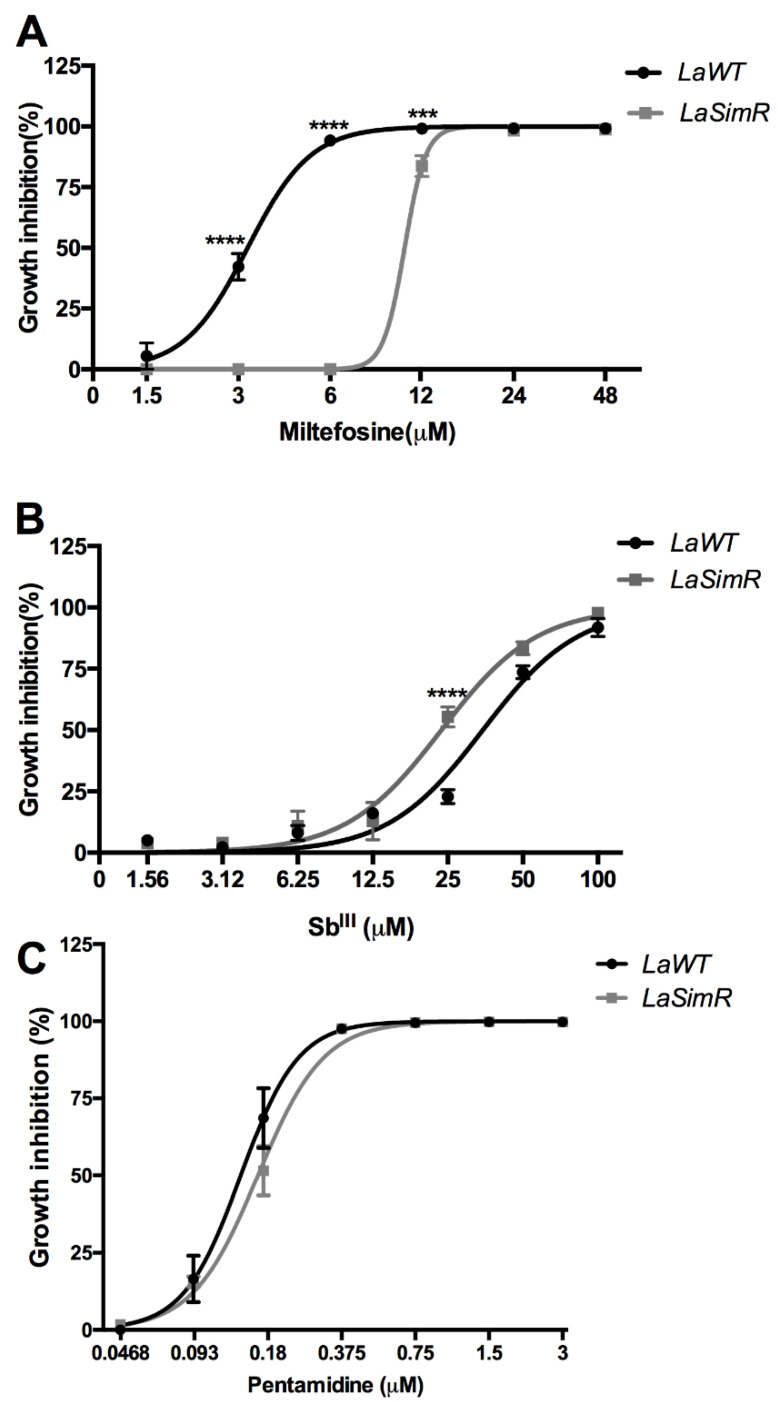
Leishmanicidal activity of antileishmanial drugs against wild-type and simvastatin-resistant strains of *L. amazonensis*. Wild-type (*La*WT) and simvastatin-resistant (*La*SimR) promastigotes were incubated at an initial concentration of 1 × 10^6^ promastigotes/mL in the presence of indicated concentrations of miltefosine (0–48 μM), trivalent antimony Sb^III^ (0–100 μM), or pentamidine (0–3 μM) for 72 h at 26 °C. After incubation, the growth was evaluated using resazurin (alamarBlue^®^); after 4 h, the reaction was evaluated by fluorimetry (excitation at 560 nm and emission at 590 nm). (**A**) Miltefosine, (**B**) Sb^III^, and (**C**) pentamidine. The experiments were performed in triplicate and repeated three times. *** *p* < 0.001; **** *p* < 0.0001.

Leishmanicidal activity of reference drugs currently used for the treatment of leishmaniasis was also assessed in the *L*aSimR line (Table 2). The resistant line was three-fold cross-resistant to miltefosine, slightly more sensitive to Sb^III,^ and equally sensitive to pentamidine when compared with the wild-type cell (Figure 5 and Table 2). Since *La*SimR was cross-resistant to ketoconazole and miltefosine, we also evaluated the possibility of efflux pumps (P-glycoproteins) participating in the resistance mechanism. To assess the presence of efflux pumps, we used the fluorochrome Rhod 123. As we can see in Supplementary Figure S4, we have not observed a difference in fluorescence between the *La*WT and *La*SimR strains. Furthermore, there is no difference in fluorescence in the parasites treated with verapamil, a drug that blocks P-glycoproteins. These results suggest that efflux pumps are unlikely to be involved in simvastatin resistance or cross-resistance to other drugs.

### 3.6. Effect of Serine Protease Inhibitors on LaWT and LaSimR

**Figure 6:**
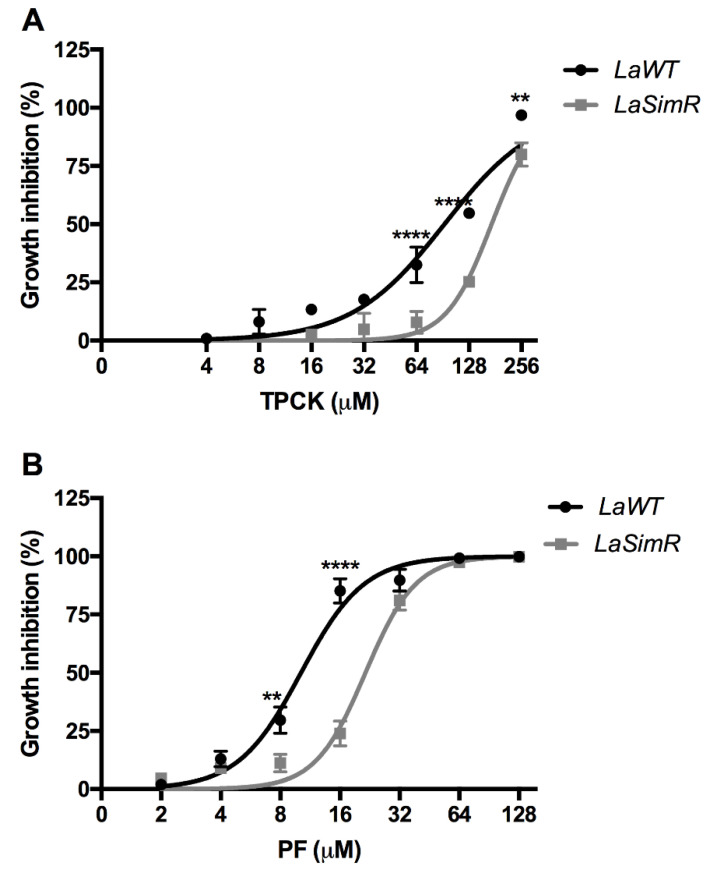
Leishmanicidal activity of serine protease inhibitors against wild-type and simvastatin-resistant strains of *L. amazonensis*. Wild-type (*La*WT) and simvastatin-resistant (*La*SimR) promastigotes were incubated at an initial concentration of 1 × 10^6^ promastigotes/mL in the presence of indicated concentrations of N-p-tosyl-L-phenylalanine chloromethyl ketone (TPCK) (0–256 μM) or PF-429242 (PF) (0–128 μM) for 72 h at 26 °C. After incubation, the growth was evaluated using resazurin (alamarBlue^®^); after 4 h, the reaction was evaluated by fluorimetry (excitation at 560 nm and emission at 590 nm). (**A**) TPCK (**B**) PF. The experiments were performed in triplicate and repeated three times. ** *p* < 0.01, **** *p* < 0.0001.

Subtilisin-encoding transcripts were preferentially accumulated in *La*SimR; hence, the parasites were treated with serine protease inhibitors to determine whether this increase is associated with resistance to these drugs (Table 2). The EC_50_ values for TPCK, a generic serine protease inhibitor, in the *La*WT and *La*SimR strains were 92.49 μM (95% CI: 77.23–110.8 μM) and 172.4 μM (95% CI: 156.5–189.9 μM), respectively (Figure 6A). The EC_50_ values for PF, a specific inhibitor of mammalian S1P, in the *La*WT and *La*SimR strains were 10.17 μM (95% CI: 9.22–11.2 μM) and 21.50 μM (95% CI: 19.91–23.21 μM), respectively (Figure 6B). These results indicated that the *La*SimR strain was cross-resistant to both TPCK and PF.

### 3.7. Effect of Tunicamycin on LaWT and LaSimR

**Figure 7:**
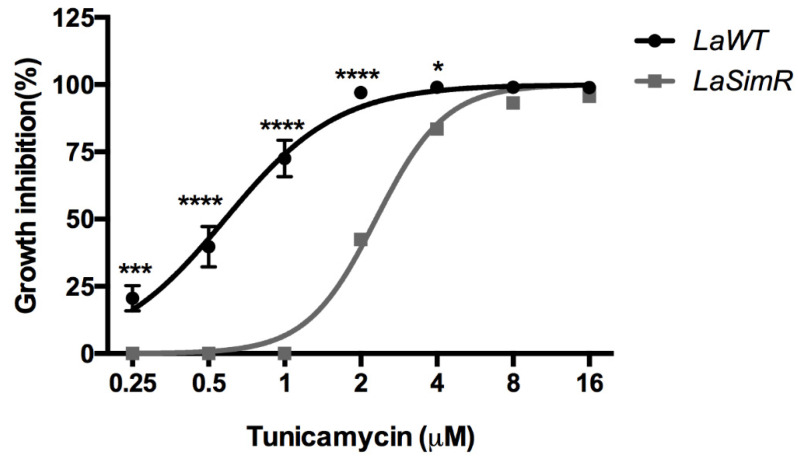
Leishmanicidal activity of tunicamycin in wild-type and simvastatin-resistant strains of *L. amazonensis*. Wild-type (*La*WT) and simvastatin-resistant (*La*SimR) promastigotes were incubated at an initial concentration of 1 × 10^6^ promastigotes/mL in the presence of indicated concentrations of tunicamycin (0–16 μM) for 72 h at 26 °C. After incubation, the growth was evaluated using resazurin (alamarBlue^®^); after 4 h, the reaction was evaluated by fluorimetry (excitation at 560 nm and emission at 590 nm). The experiments were performed in triplicate and repeated three times. * *p* < 0.05; *** *p* < 0.001; **** *p* < 0.0001.

Tunicamycin is an antibiotic that inhibits N-acetylglucosamine transferases and was used to induce the activation of another pathway dependent on S1P in mammalian cells [38].To assess whether an increase in the expression of subtilisin in the *La*SimR strain interferes with tunicamycin activity, we treated the *La*SimR and *La*WT strains with tunicamycin. The *La*SimR strain was cross-resistant to tunicamycin, with an EC_50_ of 2.29 μM (95% CI: 2.16–2.43 μM), whereas the *La*WT strain was more sensitive to this inhibitor, with an EC_50_ of 0.58 μM (95% CI: 0.51–0.65 μM) (Figure 7 and Table 2).

## 4. Discussion

Sterols are very important for the viability of eukaryotic cells. The formation of sterols, such as cholesterol and ergosterol, presumably coincided with a gradual increase in the levels of oxygen in the atmosphere during the Precambrian. This association suggests that sterols greatly contributed to the evolution of unicellular eukaryotes driven by the appearance of atmospheric oxygen, which required modifications due to the use of oxygen in the metabolic pathways of the organisms [39,40,41].

The induction of resistance in *Leishmania* spp. by pharmacological pressure with antileishmanial drugs has been used to better understand the mechanisms of resistance of the parasites, to identify new drug targets, and to determine whether the resistance is related to the regulation of gene expression [26,42,43]. In the present study, the induction of resistance was used as a pharmacological tool to determine the changes in the biology of the parasite due to stress caused by the presence of simvastatin, an HMGR inhibitor. HMGR catalyzes one committed step at the beginning of the sterol biosynthesis pathway.

Alterations in the sterol profile of *La*Sim*R* were detected in the present study. As expected, dehydroepisterol (ergosta-5,7,24-trien-3β-ol) was the most abundant sterol found in the *La*WT promastigotes. Furthermore, the levels of stigmastane-related sterols were low in *La*WT promastigotes. Literature data show that, in the amastigote form, these compounds can represent approximately 20% of sterols, depending on *Leishmania* species. This increase during parasite transformation may benefit the intracellular form [44]. We observed differences in the mRNA levels and sterol profiles at different incubation times, hence corroborating data from others. Indeed, Yao and Wilson showed that *Leishmania infantum* changes sterol composition during metacyclogenesis, indicating different quantities of sterols in the logarithmic phase and the growth of metacyclic promastigotes [45]. We did not observe the ergosta-7-24-dien-3β-ol sterol in the strain *La*WT 72 h, which is compensated by the increase in the other ergostane sterol, dehydroepisterol.

Table 1 shows that the sterol profile of the *La*SimR strain is altered in the absence of simvastatin pressure. Furthermore, when we continued with the simvastatin pressure, we observed more drastic alterations in the biosynthesis and the regulation of the sterol pathway (Figure 2). These results indicated that simvastatin alters the sterol profile but yet the parasites are still alive despite that their ergostane sterol content is depleted. Although not evaluating the sterol profile in the *La*WT strain in the presence of simvastatin (a strain that does not survive at a concentration of 75 µM), some works in the literature show that other inhibitors of the enzyme HMG-CoA reductase lead to a decrease in the ergosterol content and lead the parasites death [46,47]. In our work, we see that even without the pressure of simvastatin, ergostane sterols are reduced, showing that the *La*SimR strain probably modified its sterols during drug selection.

An increase in the relative amounts of cholestane- and stigmastane-based sterols and a decrease in sterols with ergostane skeleton were detected in *La*SimR. Maintaining the *La*SimR strain in the presence of simvastatin resulted in even greater accumulation of stigmastane-based sterol; however, the levels of ergostane-based sterols were nearly undetectable. The differences in the sterol profiles of the *La*SimR strains indicated regulation of ergosterol biosynthesis, and the presence of simvastatin further augmented these differences. The *La*SimR strain underwent reprogramming of sterol metabolism; however, it is not known if this change was due to alterations in the synthesis or consumption of sterols. Therefore, the expression of the enzymes of sterol biosynthesis, including SMT, was assessed.

SMT is responsible for the addition of the methyl group from S-adenosyl methionine (SAM) to carbon-24 of the sterol side chain during biosynthesis of ergostane- and stigmastane-based sterols [44]. A significant increase in the levels of SMT mRNA was detected in the *La*SimR strain cultivated for 48 h; however, the levels in the *La*SimR strain cultivated for 72 h were not different from that in *La*WT. Maintaining the *La*SimR strain in the presence of 75 μM simvastatin for 288 h (12 days) resulted in a substantial 80-fold increase in these levels compared with that in the *La*SimR + Sim cultivated for 72 h. SMT acts in two steps of ergosterol biosynthesis; the first step uses cholestane steroids to synthesize ergostane sterols, and these compounds are also the substrates for SMT in the synthesis of stigmastane-related sterols [48]. Therefore, an increase in SMT expression accounts for a decrease in cholestane-related sterols (cholesta-5,7,24 trien-3β-ol) and an increase in stigmastane-related sterols (Stigmasta-5,7,22–trien-3β-ol) in *La*SimR 288 + Sim in comparison to *La*SimR + Sim 72h, as revealed by GC-MS analysis. This result suggests that the levels of mRNA and SMT activity are increased in the *La*SimR mutants, converting more ergostane to stigmastane sterols.

Sterols from higher plants, fungi, and trypanosomatids differ from vertebrate sterols by the presence of an extra alkyl group at carbon-24. Biosynthesis of phytosterols in plants includes two methyltransferases involved in the biosynthesis of 24-methyl and 24-ethyl sterols. The first committed step is catalyzed by SMT1 (sterol C-24-methyltransferase); subsequently, SMT2 catalyzes methyl transfer to methylenelophenol, directing the product toward the biosynthesis of sitosterol and stigmasterol [49]. Two homologous copies of the SMT genes were identified in *L. major*, *L. donovani*, *L. infantum*, *L. mexicana*, *L. gerbilli*, and *L. aethiopica*. Knockout of two copies of the SMT genes in *L. major* (the SMT80 and SMT90 genes) and reintroduction of these genes demonstrated differences in the regulation of sterol biosynthesis by these enzymes. The absence of SMT results in total depletion of ergostane-based sterols and accumulation of cholesterol-like sterols; however, reintroduction of SMT80 alone resulted in the substantially more efficient restoration of sterol synthesis than that achieved by the reintroduction of SMT90 [50].

Furthermore, the *La*SimR strain was cross-resistant to another inhibitor of the sterol biosynthesis pathway, ketoconazole (C14DM inhibitor). This cross-resistance suggests that the mechanism of resistance to simvastatin also interferes with a downstream step of the pathway. Indeed, analysis of the mRNA levels in the *La*SimR strain indicated significant accumulation of the transcripts of C14DM, which is an enzyme of the sterol biosynthesis pathway. We previously demonstrated that induced pharmacological pressure with ketoconazole in *L. amazonensis* increases the levels of C14 mRNA and led to the suggestion that resistance to ketoconazole was associated with increased expression of the putative target enzyme [26]. These findings are consistent with our observation that *La*SimR strain is less sensitive to ketoconazole (Figure 4B) and of the three-fold higher expression of C14DM in *La*SimR (Figure 2A).

The *La*SimR strain was not cross-resistant to reference drugs against leishmaniasis, trivalent antimony, and pentamidine. These drugs are not directly related to the sterol biosynthesis pathway, suggesting that resistance induced by simvastatin is selective for the lipid biosynthesis pathway.

Although antimonial drugs do not directly act on the sterol biosynthesis pathway, studies in *L. donovani* demonstrated that parasites with lower ergosterol contents are more susceptible to antileishmanial drugs, such as potassium antimony tartrate containing trivalent antimony [51]. Parasite susceptibility to antimony was proposed to be determined by the cellular level of ergosterol [52]. Our results showed that the *La*SimR strain has lower ergostane-based sterols than *La*WT, contributing to the greater sensitivity observed in the resistant strain.

The simvastatin-resistant strain was cross-resistant to miltefosine. Studies in *L. donovani* demonstrated that miltefosine can cause major changes in lipid metabolism [53]. *Leishmania* cells selected for amphotericin B resistance and manifesting certain changes in sterols were shown to contain a mutation of miltefosine transporter and were cross-resistant to miltefosine [54,55]. Using fluorescent dyes [56,57] and inhibitors, we could not obtain evidence for efflux of drugs to explain the observed cross-resistance (Supplementary Figure S4).

Analysis of simvastatin target HMGR gene expression indicated significant accumulation of its transcripts at 48 h and 288 h in the *LaSimR* and *L*aSimR + Sim strain, respectively. Montalvetti et al. demonstrated that increased activity of HMGR is associated with the changes in the amount of HMGR protein with alteration in the sterol profile of *L. major* promastigotes treated with lovastatin. In contrast to our study, the levels of HMGR mRNA were unchanged [17].

In mammals, HMGR is regulated by cholesterol in the external environment, but in *Leishmania*, the enzyme does not seem to have this exact regulatory mechanism. A sterol-sensitive domain is present in mammalian HMGR and plays an essential role in regulating cholesterol synthesis [58,59,60,61], while this domain is absent in *Leishmania* HMGR. Then, cholesterol from serum does not seem to be a regulator of the enzyme in *Leishmania* [17]. In *T. brucei*, the cytosolic products of the mevalonate pathway act as negative regulators of HMGR. In mammalian and fungal cells, the regulation of HMGR is controlled by the levels of the sterol intermediates and cholesterol and ergosterol products, respectively [58,59,60,61]. It is possible that other ergostane-based sterols in the *La*SimR strain may be regulating the expression of HMGR.

In addition to the expression of HMGCo-A reductase, we highlighted the increase in C14DM and SMT mRNA, suggesting that their gene products participate in the regulation of sterol biosynthesis. Furthermore, using a pharmacological approach, we showed that the *La*SimR strain is cross-resistant to ketoconazole (an inhibitor of the C14DM enzyme), in line with the increased expression of the gene. Treatment of *L. major* promastigotes with ketoconazole increases the HMGR activity. It is associated with parallel changes in the amount of the HMGR protein, indicating a regulatory relationship between C14DM and HMGR [17]. In *T. cruzi* treated with simvastatin, no increase in the level of C14-demethylase transcript was observed, showing that the inhibition of HMGR is not sufficient to alter the expression [28].

Furthermore, the alteration in C14DM transcripts may participate in the changes in sterol metabolism that lead to parasites becoming resistant to simvastatin. These results reinforce the idea that the biosynthesis pathway is dynamic, where the decrease or increase in expression of an enzyme can affect other enzymes in the path and even lead to resistance to drugs that inhibit other enzymes, such as the squalene synthase, which can be related to itraconazole resistance (C14DM inhibitor) [62]. Moreover, there may be multiple sterol biosynthesis control points, and different enzymes could control the sterol biosynthesis regulation. In mammals, in addition to HMGR, the SM enzyme has been proposed as a rate-limiting enzyme in cholesterol synthesis [61].

Our results raise the hypothesis that C14DM and SMT may be related to rate-limiting enzymes in the ergosterol biosynthesis of *Leishmania*. However, the importance of C14DM for cell functions goes beyond sterol biosynthesis. *L. major* C14DM knockout leads to an increase in mRNA degradation and transcription reduction, disrupting gene expression in the defective parasite sterol production [63].

Subtilisin from *Leishmania* spp. was characterized as a processing enzyme in the regulation of the trypanothione reductase system, assisting in redox homeostasis and protecting the parasite from oxidative stress in the host macrophages [22]. Moreover, subtilisin has been shown to be essential in *L. major* [22]. The levels of SUB transcripts were increased in the *La*SimR strain, suggesting that SUB is regulated by sterol levels. We evaluated the response to oxidants in the resistant strain with hydrogen peroxide (H_2_O_2_) but found no difference between *La*WT and *La*SimR. These findings demonstrate that the increase in subtilisin transcripts alone is not enough to protect against oxidative damage (Supplementary Figure S5). The accumulation of SUB transcripts in the *La*SimR mutants led us to test their cross-resistance to the serine protease inhibitors TPCK and PF. The *La*WT strain was sensitive to TPCK and PF, and the *La*SimR strain was less sensitive to the inhibitors. Thus, the accumulation of SUB transcripts may contribute to the cross-resistance to TPCK and PF.

Tunicamycin is an antibiotic that blocks enzymes essential for glycoprotein synthesis in mammals to disrupt protein folding in the endoplasmic reticulum (ER) [64]. Accumulation of unfolded proteins in the ER results in proteolytic cleavage of ATF6, a transcription factor bound to the ER membrane, to release the N-terminal domain that enters the nucleus [65]. Proteolytic cleavage of ATF6 is catalyzed by the S1P and S2P enzymes, and the same enzymes process sterol regulatory element-binding protein (SREBP). In the nucleus, cleaved ATF6 activates the transcription of at least three genes, GRP78, GRP94, and calexin, encoding chaperone proteins that restore protein folding in the ER lumen [38]. Tunicamycin was shown to inhibit the growth and infectivity of some *Leishmania* species, such as *L. amazonensis* [66], and tunicamycin induces programmed cell death in *L. major* [67]; however, the exact mechanism of action of the drug is unknown. Hence, the *La*WT strain was hypersensitive to this antibiotic. Subtilisin transcripts accumulated in the simvastatin-resistant strain and the cholesterol deprivation (Figure 3), possibly similar to mammalian S1P. *Leishmania* subtilisin may be involved in the mechanisms of gene regulation in *Leishmania*; however, additional experiments are necessary to prove this hypothesis.

The sterol biosynthesis pathway in *Leishmania* spp. has been extensively studied as a pharmacological target for the treatment of leishmaniasis. Studies of the regulation of this pathway are very important for expanding our knowledge of the biology of this parasite and for improving the treatment of this disease. In the present study, we produced a strain of *L. amazonensis* resistant to simvastatin, which was used as a tool for providing additional information about the biology of this protozoan [68], including regulation of the biosynthesis pathways of sterols and subtilisin. Additional studies should investigate the mechanism of this regulation.

In summary, *La*SimR was resistant to ergosterol pathway inhibitors, antileishmanial drug miltefosine, serine peptidase inhibitors, and tunicamycin, an inhibitor of glycoprotein synthesis but not to Sb^III^ and pentamidine. We observed upregulation of C14DM and SMT, the enzymes of the sterol biosynthesis pathway that can be associated with an increase in cholestane and stigmastane sterols detected in this strain. We also provide for the first time evidence for a cross-talk between the sterol pathway and serine peptidase subtilisin.

## Figures and Tables

**Table 1:** Analysis of the sterol profiles of promastigotes of *L. amazonensis La*WT and *La*SimR.

Sterols	MW	Relative Amount (%)					
		*La*WT 48 h	*La*SimR 48 h	*La*WT 72 h	*La*SimR 72 h	*La*SimR 72 h + Sim	*La*SimR 288 h + Sim
(1) Cholesterol	386	5.06 ± 1.4	5.56 ± 1.5	8.23 ± 3.03	8.62 ± 3.94	5.33 ± 1.31	4.62 ± 1.68
(2) Cholesta-7,24–dien-3β-ol	384	0.96 ± 0.14	nd	nd	nd	nd	nd
(3) Stigmasta-5, 22–dien-3β-ol (Stigmasterol)	412	nd	1.17 ± 0.58	2.83 ± 1.11	13.89 ± 2.38	16.42 ± 2.66	13.38 ± 4.7
(4) Cholesta-5,7,24–trien-3β-ol	382	12.85 ± 2.16	29.62 ± 1.05 (***)	13.5 ± 4.21	30.16 ± 8.83 (#)	43.51 ± 8.3	31.1 ± 3.20 (&)
(5) Stigmasta-5,7,22–trien-3β-ol	410	0.33 ± 0.18	3.3 ± 1.65	3.89 ± 1.78	28.45 ± 3.22 (###)	31.43 ± 6.65	50.9 ± 2.49 (ππ) (&&)
(6) Ergosta-5,7,24-trien-3β-ol (dehydroepisterol)	396	77.8 ± 4.8	59.31 ± 1.4 (**)	71.55 ± 4.57	18.88 ± 1.14 (###)	3.31 ± 3.1 (π)	nd (ππ)
(7) Ergosta-7,24-dien-3β-ol (episterol)	398	3.0 ± 1.17	1.04 ± 0.8	nd	nd	nd	nd

MW, molecular weight (Da); nd, not detected. Symbols indicate significant differences between *La*WT 48 h vs. *La*SimR 48 h (** *p* < 0.01; *** *p* < 0.001), *La*WT 72 h vs. *La*SimR 72 h (# *p* < 0.05; ### *p* < 0.001), *La*SimR 72 vs. *La*SimR + Sim 72 h and *La*SimR 72 h vs. *La*SimR + Sim 288 h (π *p* < 0.05; ππ *p* < 0.01), *La*SimR + Sim 72 h vs. *La*SimR + Sim 288 h (& *p* < 0.05; && *p* < 0.01).

**Table 2:** Sensitivity of *LaWT and LaSimR* promastigotes to antileishmanial drugs.

Drugs	EC_50_ (μM)	
	*La*WT	*La*SimR
Simvastatin	39.72 (33.47–47.15)	90.04 (85.46–94.87)
Ketoconazole	12.43 (11.84–13.05)	20.76 (19.29–22.34)
Terbinafine	29.77 (27.79–31.89)	23.90 (22.78–25.08)
Miltefosine	3.21 (3.10–3.33)	10.53 (5.0–22.16)
Sb^III^	34.56 (31.33–38.12)	23.57 (21.24–26.16)
Pentamidine	0.15 (0.13–0.16)	0.17 (0.16–0.18)
PF-429242	10.17 (9.22–11.2)	21.50 (19.91–23.21)
TPCK	92.49 (77.23–110.8)	172.4 (156.5–189.9)
Tunicamycin	0.58 (0.51–0.65)	2.29 (2.16–2.43)

## Data Availability

Date included in the manuscript are available from the corresponding author.

## Supplementary Materials

The following are available online at https://www.mdpi.com/article/10.3390/microorganisms10020398/s1, Figure S1: Thin layer chromatography (TLC) of FBS and FBSd. Figure S2: Leishmanicidal activity of simvastatin in the wild-type and simvastatin-resistant strains of *L. amazonensis*. Figure S3: Growth curve of *L. amazonensis* promastigotes. Figure S4: Rhod-123 efflux in *La*WT and *La*SimR promastigotes observed by Flow Cytometry. Figure S5: Growth inhibition of *L*aWT and *La*SimR promastigote exposed to hydrogen peroxide (H_2_O_2_).
