# Supplementary material for: Simvastatin Resistance of Leishmania amazonensis Induces Sterol Remodeling and Cross-Resistance to Sterol Pathway and Serine Protease Inhibitors
Source: Microorganisms. 2022 Feb 9;10(2):398. doi: 10.3390/microorganisms10020398 (PMC8877030; doi:10.3390/microorganisms10020398)
Supplement: Supplementary file 1 [file microorganisms-10-00398-s001.zip › microorganisms-1441249-supplementary.pdf]

**Simvastatin resistance in *Leishmania amazonensis* induces sterol remodeling and cross-resistance to sterol pathway and serine protease inhibitors**

Thais Tenorio Soares Fujii[1#], Pollyanna Stephanie Gomes[1,3#], Rubens Lima do Monte-Neto[4], Daniel Claudio de Oliveira Gomes[5], Marc Ouellette[6], Eduardo Caio Torres-Santos[7], Valter Viana Andrade-Neto[7*], Herbert Leonel de Matos Guedes[1,2,3*]

***<u>Affiliations</u>***

[1]Laboratório de Imunofarmacologia, Instituto de Biofísica Carlos Chagas Filho (IBCCF), Universidade Federal do Rio de Janeiro (UFRJ), Rio de Janeiro 21941-901, Brazil; thaistenoriosoares@gmail.com

[2]Laboratório de Imunobiotecnologia, Instituto de Microbiologia Paulo de Góes Universidade Federal do Rio de Janeiro, Rio de Janeiro 21941-901, Brazil; pollyannaufrj@gmail.com

[3]Laboratório Interdisciplinar de Pesquisas Médicas, Instituto Oswaldo Cruz, Fundação Oswaldo Cruz- Fiocruz, Rio de Janeiro 21040-900; Brazil;

[4] Grupo de Pesquisas em Biotecnologia Aplicada a Patógenos- Instituto René Rachou, Fundação Oswaldo Cruz-Fiocruz Minas, Belo Horizonte, Minas Gerais 30190-002, Brazil; rubens.monte@fiocruz.br

[5] Núcleo de Doenças Infecciosas, Universidade Federal do Espírito Santo, Vitória, Espírito Santo 29075-910, Brazil; dgomes@ndi.ufes.br.

[6]CHU de Quebec Research Center, Division of Infectious Disease and Immunity, Quebec, QC, Canada, Department of Microbiology, Infectious Disease and Immunology, Laval University, Quebec City, G1V 4G2 Canada; marc.ouellette@crchudequebec.ulaval.ca

[7]Laboratório de Bioquímica de Tripanossomatídeos, Instituto Oswaldo Cruz, Fundação Oswaldo Cruz- Fiocruz, Rio de Janeiro 21040-900, Brazil; ects@ioc.fiocruz.br

[#]Both authors contribute equally to this work.

*Senior authors and authors of correspondence:

## Supplementary Figures

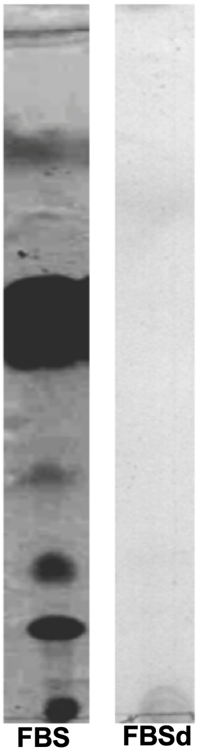


**Supplementary Figure S1.** Thin layer chromatography (TLC) of FBS and FBSd. Fetal bovine serum **(**FBS) aliquots containing 0.1 mg/mL EDTA were added to 10 mL butanol-DIPE (diisopropyl ether) (40:60, v/v) for delipidation. The delipidated FBS (FBSd) obtained was then sterilized and dissolved in chloroform to be analyzed by TLC on a silica plate. The plate was previously impregnated with silver nitrate (1%). The plate was run in two steps. The first run was performed with hexane:ethyl ether:acetic acid (60:40:1, v/v), and the second was performed with hexane:chloroform:acetic acid (80:20:1, v/v). The plates were developed using a charring reagent ($CuSO_4$) followed by heating at 200 °C for 20 min

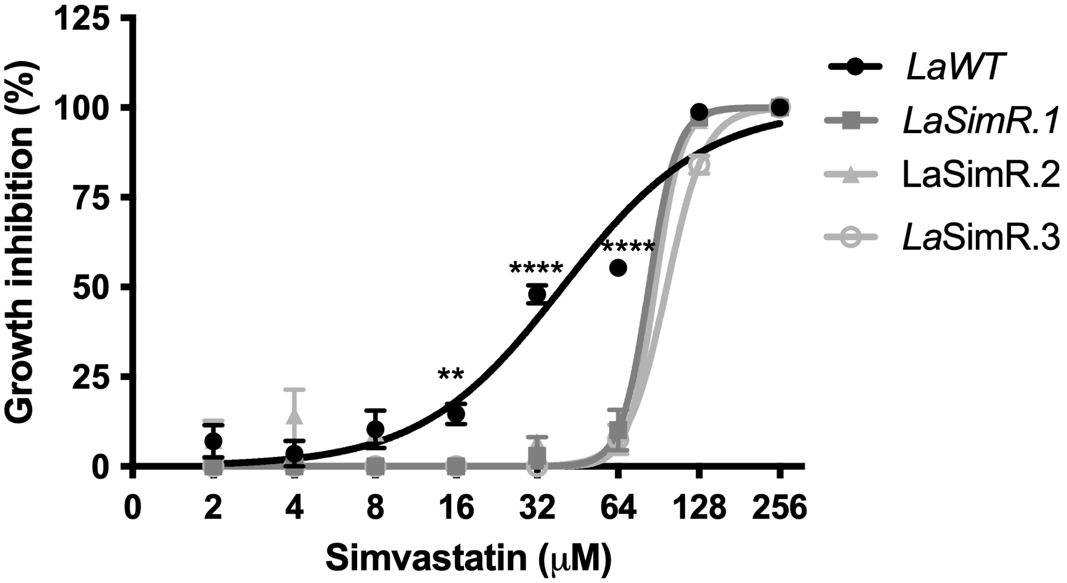


**Supplementary Figure S2.** Leishmanicidal activity of simvastatin in the wild-type and simvastatin-resistant strains of *L. amazonensis*. Wild-type (*La*WT) and simvastatin-resistant (*La*SimR.1, *La*SimR.2, *La*SimR.3) promastigotes were incubated at an initial concentration of 1 x $10^6$ promastigotes/mL in the presence of various concentrations of simvastatin (0-256 μM) in 96-well plates for 72 h at 26 °C. After incubation, the growth was evaluated using resazurin (alamarBlue®); after 4 h, the reaction was evaluated by fluorimetry (excitation at 560 nm and emission at 590 nm). The experiments were performed in triplicate and repeated three times. **P<0.01; **** P<0.0001.

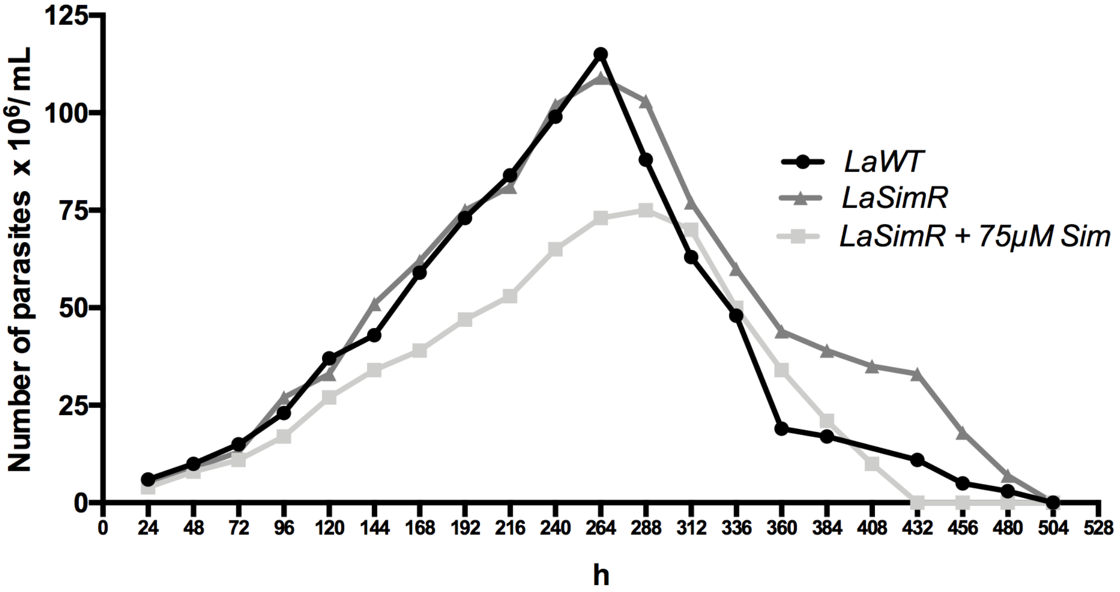


**Supplementary Figure S3. Growth curve of *L. amazonensis* promastigotes.** The evaluation of parasites growth of *La*WT, *La*SimR, *La*SimR + 75μM Sim was determined by counting in a Neubauer camera each 24 h for 528 hours.

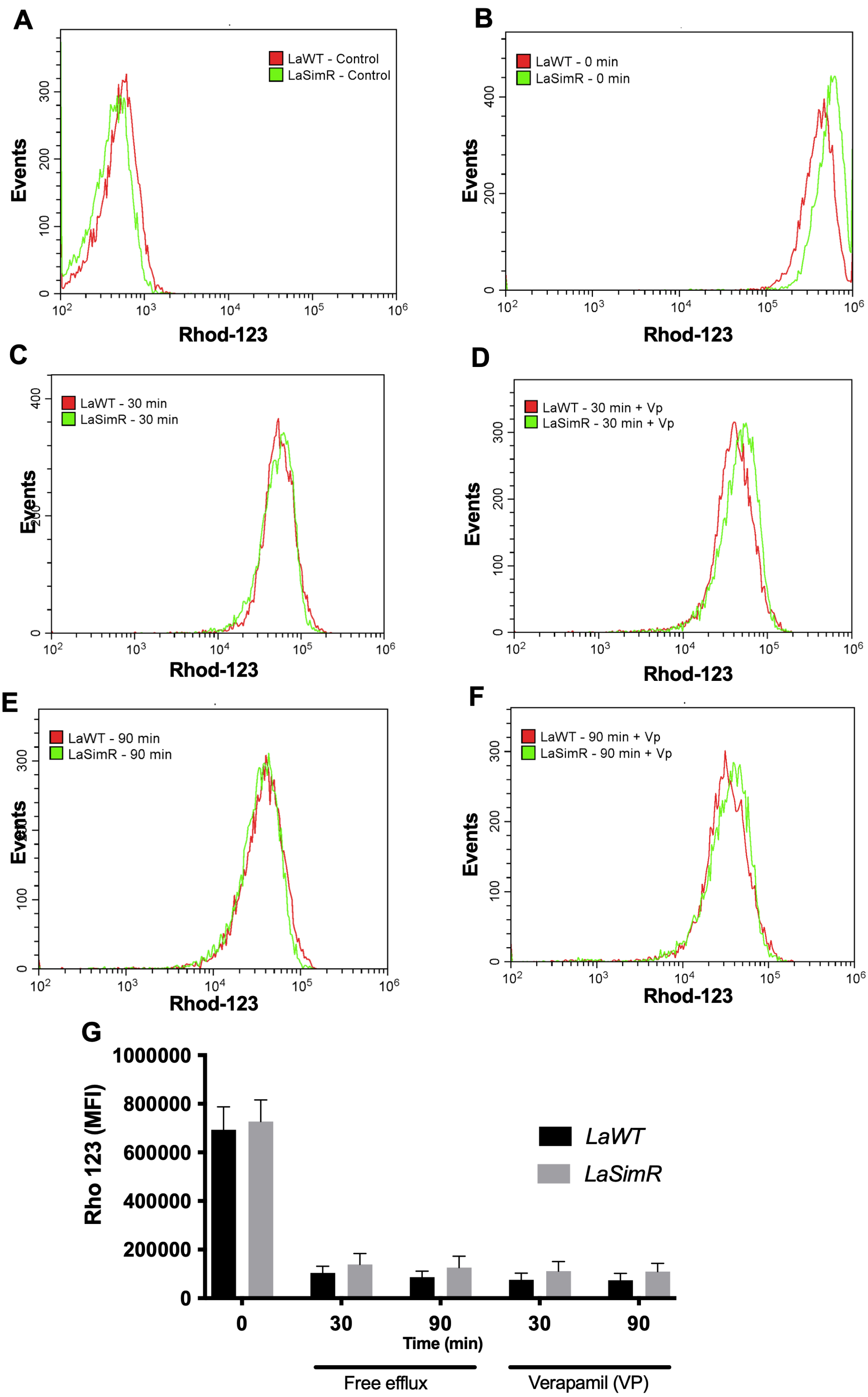
A
LaWT - Control
LaSimR - Control
Events
Rhod-123
B
LaWT - 0 min
LaSimR - 0 min
Events
Rhod-123
C
LaWT - 30 min
LaSimR - 30 min
Events
Rhod-123
D
LaWT - 30 min + Vp
LaSimR - 30 min + Vp
Events
Rhod-123
E
LaWT - 90 min
LaSimR - 90 min
Events
Rhod-123
F
LaWT - 90 min + Vp
LaSimR - 90 min + Vp
Events
Rhod-123
G
Rho 123 (MFI)
1000000
800000
600000
400000
200000
0
LaWT
LaSimR
0
30
90
30
90
Time (min)
Free efflux
Verapamil (VP)

**Supplementary Figure S4. Rhod-123 efflux in *La*WT and *La*SimR promastigotes observed by Flow Cytometry.** Promastigotes of *La*WT and *La*SimR were incubated in RPMI medium in the presence and the absence of 100 μM of verapamil hydrochloride (Vp) for 1 hour at 26 °C. After this time, parasites were incubated in the presence and the absence of Rhodamine-123 (Rhod-123) (5 μg/ml) for 30 min, washed three times, resuspended in 1 ml PBS buffer, and incubated in the present and the absence of Vp for 30 min and 90 min for observed Rhod-123 efflux. Data analysis was performed using the CytExpert software. **(A)** negative control (No Rhod123), **(B)** positive control (0 min of Rhod-123 efflux) **(C)** 30 min of Rhod-123 efflux, **(D)** 30 min of Rhod-123 efflux + Vp, **(E)** 90 min of Rhod-123 efflux, **(F)** 90 min of Rhod-123 efflux + Vp, **(G)** Median Fluorescence Intensity (MFI). The assays were performed in triplicate, and the experiments were repeated three times.

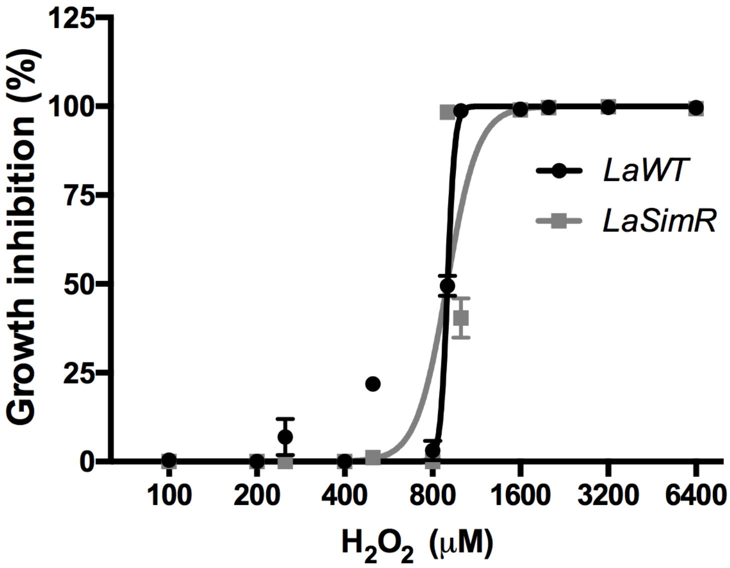


**Supplementary Figure S5. Growth inhibition of *La*WT and *La*SimR promastigote exposed to hydrogen peroxide ($H_2O_2$).** *L. amazonensis* promastigotes *La*WT and *La*SimR were incubated at 26 °C for 72 h in RPMI and exposed to $H_2O_2$ (0-6400 µM). Parasite viability was assessed using 50 µM resazurin (alamarBlue®) for 4 h at 26 °C. The reaction product was measured by fluorimetry (SpectraMax Gemini XPS, Molecular Devices, CA, USA) (excitation at 560 nm and emission at 590 nm). The assays were performed in triplicate, and the experiments were repeated three times.
